# Digital Health Technologies for Parental Education in Neonatal Intensive Care Units: Current Evidence and Implications for Nursing Practice

**DOI:** 10.7759/cureus.108974

**Published:** 2026-05-16

**Authors:** Aslinur Taskin Guzelyazici, Evrim Kiziler

**Affiliations:** 1 Department of Midwifery, Pediatric Nursing, Ankara Medipol University, Ankara, TUR; 2 Department of Nursing, Ankara Yıldırım Beyazıt University, Ankara, TUR

**Keywords:** family-centered care, mobile applications, neonatal intensive care units (nicu), parent education, self efficacy, telemedicine

## Abstract

The neonatal intensive care unit (NICU) is a highly complex and emotionally challenging clinical environment, and this situation can make it difficult for parents to interact with their infants and to adapt to the learning process. Parents are often expected to understand complex medical information in a short time, while also coping with emotional stress. In practice, traditional face-to-face education is often insufficient, mainly due to time limitations, differences in how information is delivered, and varying levels of parental health literacy. For this reason, digital health technologies have increasingly started to be used as supportive tools in recent years.

In this review, we aimed to explore how digital health technologies are used for parent education in NICUs, and to evaluate their possible effects on both parents and infants. At the same time, we also focused on what these developments may mean for nursing practice. A narrative literature review was conducted using PubMed, Google Scholar, and Semantic Scholar databases (2015-2025). Relevant literature on this topic was reviewed using commonly used academic databases and key search terms related to NICU, telemedicine, parent education, and mobile applications. The included studies were then examined using a thematic approach.

According to the findings, a range of digital tools are currently being used in NICU settings, including videoconferencing systems, webcams, mobile applications, web-based platforms, and messaging services. Many studies suggest that these tools may help reduce parental anxiety, increase satisfaction, and support parent-infant bonding. However, the results are not completely consistent. It appears that the effectiveness of these technologies depends largely on how they are implemented in clinical practice. In addition, some concerns have been raised, especially regarding the reliability of certain mobile applications and possible patient safety risks in poorly organized telemedicine use.

Overall, digital health technologies seem to be most helpful when they are used alongside nurse-led care rather than as a replacement for it. Adequate training, manageable workloads, and equal access for families also appear to be important factors. These findings suggest that careful and well-planned implementation is necessary and that nurses continue to play a key role in supporting parents through digital education in NICUs.

## Introduction and background

One of the most emotionally taxing events a family may go through is caring for a newborn in the neonatal intensive care unit (NICU). Parents of hospitalized newborns must rapidly understand their child’s medical condition, assume specific caregiving responsibilities, and adapt to a significant shift in identity from expectant parents to primary caregivers of a physically vulnerable infant. This transition is often accompanied by increased anxiety, emotional distress, and feelings of helplessness. In addition, maintaining parent-infant closeness while coping with necessary medical separation presents a significant emotional and relational challenge in NICU settings [1]. If not adequately addressed, these reactions may negatively affect parental well-being and, consequently, infant outcomes [2,3].

Family-centered neonatal care strongly relies on effective parental education. Recent systematic reviews have identified key components of effective parenting education interventions for parents of preterm infants in NICUs, emphasizing structured, accessible, and family-centered approaches [4,5]. Well-informed parents are more likely to breastfeed, actively participate in caregiving, and feel better prepared for the transition to home [6]. In NICUs, parent education has traditionally been delivered by nurses through face-to-face interactions and printed materials. Although these approaches are effective, they have limitations, including restricted access to information outside clinical encounters, variability in content delivery, and increased nursing workload.

Digital health technologies, including mobile applications, telemedicine systems, videoconferencing platforms, and electronic educational tools, are increasingly being used to support healthcare delivery, communication, and parent education in NICU settings. These technologies also include webcams, electronic health record (EHR)-linked portals, and messaging systems. They enable parents to access information more flexibly, maintain connection with their infants, and communicate with healthcare professionals beyond physical presence in the NICU. The COVID-19 pandemic further accelerated this shift, as visitation restrictions led to the rapid integration of telemedicine into routine neonatal care [7].

Despite the increasing adoption of digital health technologies in NICUs, the existing evidence remains heterogeneous and fragmented. Additionally, recent research trends indicate growing interest in eHealth-supported interventions aimed at improving parental and infant outcomes during the transition from NICU to home [8]. Studies differ in terms of the technologies evaluated, the type and scope of educational content provided, targeted outcomes, and methodological approaches. Moreover, nursing-related dimensions, such as workflow integration, training needs, and the quality of nurse-parent interactions, are not consistently addressed.

Given the heterogeneity of study designs, interventions, and outcomes in this field, a narrative review approach was considered appropriate. In this context, it is important to clarify that this review focuses on digital health technologies that support parental education, engagement, and communication within NICU settings. Therefore, this narrative review aims to provide a comprehensive synthesis of digital health technologies used to support parents in NICUs, evaluate their effects on parental and infant outcomes, explore their acceptability, and identify implications for nursing practice [3,9].

## Review

Methods

Design and Review Approach

This study was conducted as a narrative literature review. A formal systematic review protocol was not applied; however, efforts were made to ensure a transparent and structured approach to literature identification, selection, and synthesis.

Search Strategy

Relevant studies published between 2015 and 2025 were identified through searches in PubMed, Google Scholar, and Semantic Scholar. The search strategy was based on combinations of keywords, including “neonatal intensive care unit (NICU),” “digital health,” “telemedicine,” “parent education,” “mobile applications,” and “family-centered care.” Additional related terms were used where appropriate to broaden the scope of the search. Reference lists of relevant articles were also screened manually to identify additional studies related to parental education, engagement, and communication in NICU settings.

Eligibility Criteria

Studies were included if they focused on the use of digital health technologies in NICU settings, addressed parent education, engagement, or communication, and were published in English between 2015 and 2025. Both original research articles and review studies were considered to provide a comprehensive overview of the topic.

Studies were excluded if they were not related to NICU settings, did not involve digital health interventions, or did not address parental outcomes or education.

Study Selection Process

The study selection process was conducted in a stepwise manner. Titles and abstracts were first screened for relevance. Potentially eligible studies were then reviewed in full text when available. In cases where full-text access was limited, available summaries were considered. The final selection was based on relevance to the review aim and scope. Studies focusing more broadly on telemedicine or digital communication were included only when their findings were considered relevant to parental education, engagement, or communication within NICU settings. A total of 25 studies were included in the final narrative synthesis.

Data Extraction and Synthesis

Data from the selected studies were examined using a thematic approach. Studies were grouped into key categories, including types of digital interventions, parent-related outcomes, infant-related outcomes, and implications for nursing practice.

Given the heterogeneity of study designs, interventions, and outcomes, a narrative synthesis approach was adopted rather than quantitative pooling.

Reporting Considerations

Although this review was not conducted as a systematic review, elements of Preferred Reporting Items for Systematic Reviews and Meta-Analyses (PRISMA)-style reporting were considered to improve transparency, particularly in describing the search process and study selection. Due to the narrative design of the review and the heterogeneity of the included studies, a formal risk of bias assessment or standardized quality appraisal was not performed.

Results

Table 1 summarizes the characteristics and main findings of the studies included in this narrative review.

**Table 1:** Summary of Included Studies on Digital Health Technologies in NICU Parent Education and Support

Author / Year	Study Design	Digital Technology	Population	Main Outcomes	Key Findings
Feeley et al., 2016 [1]	Qualitative study	Virtual communication support	NICU nurses	Parent-infant closeness, communication	Nurses emphasized the importance of maintaining parent-infant connection despite physical separation.
Epstein et al., 2017 [2]	Integrative review	Multiple digital communication technologies	Parents of NICU infants	Communication, parental support	Digital technologies improved communication and parental engagement.
Kim & Hong, 2025 [3]	Systematic review	Health literacy interventions	Parents of preterm infants	Health literacy, education outcomes	Structured educational interventions improved parental knowledge and understanding.
O’Brien et al., 2018 [4]	Cluster randomized trial	Family integrated care model	NICU families	Parent and infant outcomes	Family-centered involvement improved parental confidence and infant outcomes.
Lubbe et al., 2026 [5]	Systematic review	Parenting education interventions	Parents of preterm infants	Education effectiveness	Accessible and family-centered educational approaches were most effective.
Sunshine et al., 2023 [6]	Narrative review	Mobile applications	NICU parents transitioning home	Parent support, discharge transition	Mobile apps supported education and transition to home care.
Lapcharoensap et al., 2021 [7]	Review article	Telemedicine	Neonatal care providers and families	Communication, remote care	Telemedicine expanded rapidly during the COVID-19 period.
Ferraz et al., 2024 [8]	Systematic review protocol	eHealth interventions	Parents of premature infants	Parent and infant outcomes	eHealth interventions may support transition from NICU to home.
Paleardi et al., 2025 [9]	Scoping review	eHealth interventions	Parents of preterm infants	Well-being, support	eHealth interventions showed potential psychological benefits.
Dunham & Marin, 2022 [10]	Scoping review	Virtual visitation	NICU parents and nurses	Parent experience, staff perceptions	Virtual visitation improved family connection but increased nursing workload concerns.
Dol et al., 2017 [11]	Systematic review protocol	eHealth interventions	NICU parents	Parent outcomes	eHealth interventions may improve parental engagement and education.
Siani et al., 2017 [12]	Scoping review	Parent-targeted eHealth	NICU parents	Parent and infant outcomes	eHealth interventions were associated with improved parental confidence and satisfaction.
Pajak et al., 2023 [13]	Systematic review	Live-streaming webcams	NICU parents	Parent well-being	Webcam systems improved reassurance but occasionally increased distress.
Gibson & Kilcullen, 2020 [14]	Review article	Web-camera systems	NICU parents	Parent-infant attachment	Webcam access supported parent-infant bonding.
Schwartz et al., 2025 [15]	Narrative review	Digital bonding technologies	Parents separated from infants	Bonding, emotional support	Technology may support bonding during separation at birth.
Setiyawati, 2023 [16]	Literature review	Video-messaging services	NICU parents	Communication	Video-messaging improved parental connection and communication.
Balasundaram et al., 2022 [17]	Quality improvement study	Technology-supported discharge education	NICU families	Parent satisfaction, discharge preparedness	Digital education improved discharge satisfaction and preparedness.
Riskin et al., 2022 [18]	Review and observational study	Digital communication during COVID-19	NICU parents	Communication, family support	Telecommunication tools became essential during pandemic restrictions.
Wang & He, 2024 [19]	Meta-analysis	eVisit technology	Parents of NICU infants	Anxiety, satisfaction	eVisit technologies reduced parental anxiety and improved satisfaction.
Kermani et al., 2023 [20]	Systematic review	Mobile applications	Parents of premature infants	App quality, outcomes	Many commercially available apps had quality limitations.
Wagenaar et al., 2024 [21]	Scoping review	Telemedicine	NICU parents	Well-being	Telemedicine may improve parental well-being during neonatal care.
Norris & Al-Muzaffar, 2020 [22]	Literature review	eHealth technologies	NICU parents	Communication	eHealth technologies supported communication during COVID-19 restrictions.
Richardson et al., 2019 [23]	Systematic app review	Mobile applications	NICU parents	App quality	Many apps lacked adequate reliability and quality standards.
Kwarteng et al., 2025 [24]	Retrospective cohort study	Telemedicine	NICU families	Healthcare utilization, safety	Telemedicine implementation was associated with missed clinical measurements.
Buccione et al., 2025 [25]	Scoping review protocol	eHealth transitional care programs	Families of preterm infants	Transitional care outcomes	Digital transitional care interventions may support family-centered care.

Types of Digital Health Technologies

The findings obtained from the included studies were organized under five main categories: videoconferencing and virtual visitation, live-streaming webcams, mobile applications, web-based platforms integrated with electronic health records, and messaging services.

Videoconferencing and Virtual Visitation

Several studies have evaluated videoconferencing platforms including Skype, FaceTime, and videophone systems as tools for maintaining maternal bonding with NICU babies [2]. These platforms enabled real-time communication, allowed parents to participate in bedside rounds remotely, and provided regular updates when parents could not be physically present [2,9-11]. Dedicated virtual visitation systems such as NICView and AngelEye were developed specifically to bridge the gap between hospitalized infants and their families [2,12].

Live-Streaming Webcams

Webcam technology that enables continuous remote viewing of babies featured prominently across a number of reviews [12-15]. Some commercial systems also allowed extended family members to view the child, which parents consistently found meaningful [12]. Unlike videoconferencing, webcams function as passive monitoring tools that allow parents to observe their infants remotely without direct interaction, which has both advantages and limitations.

Mobile Applications

Mobile applications were among the most frequently studied digital technologies [6,10]. Mobile applications developed for parents of NICU infants offer features such as educational content, journaling, and infant growth tracking [6]. However, concerns remain regarding the quality and reliability of some applications. This represents an important concern for clinical practice.

Web-Based Platforms and EHR-Integrated Portals

Online educational platforms provided structured, self-paced learning environments for parents [3,9]. One quality improvement initiative integrated discharge education content directly into the hospital’s EHR-linked outpatient portal, allowing families to access and revisit educational materials at their convenience, while nursing staff remained available to reinforce key topics when needed [16].

Messaging Services

Text messaging and SMS were used for asynchronous communication between families and clinical teams [2,3]. One pilot study used WhatsApp to send parents short daily video updates and voice messages about their child’s condition, offering a more personal communication channel than traditional phone calls, although communication remained asynchronous in nature [17].

Parent-Related Outcomes

Psychological well-being: Anxiety and stress reduction were among the most commonly evaluated outcomes in the reviewed studies. A meta-analysis including eight studies and data from 1,450 parents reported that electronic visitation technologies were associated with significantly lower maternal anxiety compared with conventional visitation (MD = -5.04; 95% CI: -5.92 to -4.17; p < 0.01) [18]. Parents who had access to webcam technology also reported lower stress levels [12,15], and improvements in parental perceptions related to having an infant in the NICU were observed following the use of Skype or FaceTime (p < 0.001 and p = 0.04) [2]. However, the findings were not entirely consistent. While many parents described live-streaming technology as reassuring [12], others reported distress when witnessing difficult situations through the camera without being able to intervene or communicate directly [12,14]. Feelings of guilt, helplessness, and emotional distress were also reported among some parents using webcam technology [14].

Self-efficacy and confidence: Digital tools appeared to strengthen parental confidence in several ways. Among parents who used mobile applications, improvements in self-efficacy scores on the Parenting Sense of Competence Scale appeared to increase with greater engagement in the intervention [7]. Parents using videoconferencing also reported feeling more confident in their caregiving role [2]. In addition, a scoping review of 10 studies found that eHealth interventions were associated with increased parental confidence and improved knowledge acquisition overall [19]. Technology-based educational interventions also contributed to improved discharge preparedness [20,21].

Knowledge and learning: Several studies reported improvements in parental knowledge and learning outcomes. In one digital intervention, maternal knowledge scores increased from 60.9 to 91.8, and follow-up attendance improved significantly (80%; p = 0.008) [3]. EHR-integrated platforms that allowed parents to revisit educational content at their own pace were considered particularly beneficial for information retention [16]. Videoconferencing-based updates also improved parental understanding, with statistically significant improvements observed in two of four subscales of a validated maternal understanding measure [2]. Parents consistently identified medical information as one of the areas in which they most wanted additional support and education [19].

Satisfaction: Satisfaction outcomes were generally positive across the included studies. A meta-analysis reported a significant improvement in hospitalization satisfaction associated with electronic visitation technologies (RR = 1.09; 95% CI: 1.05-1.13; p < 0.01) [18]. Maternal satisfaction also improved significantly following internet-based education in one quasi-experimental study (p < 0.001) [19]. In a quality improvement initiative using an EHR-linked portal, discharge preparedness satisfaction scores increased from 47% to 70%, and 92% of families rated the digital education approach highly during post-discharge follow-up [16]. WhatsApp-based updates were also positively received in terms of communication experience, although overall satisfaction scores did not differ significantly from those of the control group [17].

Infant-Related Outcomes

The evidence on infant outcomes was considerably more limited and less consistent than that for parent outcomes. The meta-analysis found no meaningful reduction in NICU length of stay associated with electronic visitation technology (MD = -1.07; 95% CI (-5.39, 3.25); p = 0.63) [18], and a systematic review by Dol et al. reached a similar conclusion, indicating that eHealth interventions had an unclear impact on neonatal outcomes, generally based on low to very low-quality evidence [11].

One narrative review reported shorter NICU stays associated with mobile app use in a subset of studies [6], although the absence of confounder adjustment limits the strength of this finding. Digital education appeared to have a more noticeable impact in the post-discharge period. Two studies found that families who received eHealth-supported education visited emergency departments less frequently after discharge, suggesting that the benefits of parent education may become more evident once parents are the primary decision-makers for their child's care [22].

Technology Acceptability

Parents generally responded well to digital tools regardless of the modality. In the videoconferencing feasibility study, more than 90% of parents rated the technology as reliable and easy to use, and around 80% reported that video and audio quality was excellent or good [2]. Among 19 parents who participated in a needs assessment for a proposed mHealth app, 17 valued the concept and 10 indicated that they would definitely use it [21].

Tablet-based discharge education received high ratings from 92% of families, and 62% of parents in one study reported that they preferred video-based hand hygiene instruction over receiving it in person from a staff member [16].

Healthcare providers, including nurses, were also highly accepting. More than 90% of clinicians found videoconferencing reliable and feasible in practice [2], and nurses who were involved in co-developing digital programs tended to view them positively [2,16].

Findings were less favorable, however, when it came to virtual visitation specifically. A scoping review focusing on nurses' experiences found generally negative perceptions, with concerns centered on increased workload, the time required to manage cameras, privacy issues, and worries about legal liability [10].

A systematic review of commercially available mobile applications added another important perspective. Although parental attitudes toward digital tools were generally positive, formal quality assessments showed that fewer than half of the available applications met acceptable quality standards, and 72% were found to be unreliable [23]. This gap between perceived usefulness and actual quality represents an important practical concern that healthcare institutions need to address.

Nursing Role and Workflow Implications

Workload changes: Nurses described a range of additional tasks that came with digital technology adoption, including managing cameras, responding to technology-related questions from parents, and completing digital check tools for outcome tracking [10,12]. Sending daily video updates via messaging platforms represented a new daily responsibility in at least one study [17], and developing content for EHR-integrated platforms required a significant upfront time investment [16]. On the other hand, formalized video-based education appeared to reduce the need for repetitive one-on-one teaching [16], and asynchronous digital platforms had the potential to reduce redundant educational tasks [21].

Nurse perceptions: Nursing views on digital technologies were somewhat mixed. Most nurses found videoconferencing workable and reliable, and many saw it as a tool that supported safe, high-quality care [2]. High family satisfaction scores in digital quality improvement initiatives suggested that nurses were engaging well with these tools [16].

However, nurses working with webcam-based virtual visitation reported generally negative experiences. They described concerns about constant observation, managing heightened parental expectations, workflow disruption, and the possibility of distressing events being captured on camera [10,12]. Several nurses also felt that reliance on webcam technology was eroding direct interaction and trust between staff and families [12].

Training and skill needs: Each technology category brought with it new skill demands. Nurses who were less confident with technology needed additional support [2], and managing webcam systems, navigating privacy requirements, and assessing parental digital literacy all emerged as competencies that nurses needed but were rarely formally trained for [7,12].

Strikingly, none of the included studies described formal nursing training protocols, including their content, duration, or how competency was assessed [2,16]. This represents a significant gap in the implementation literature.

Role evolution: There were signs across the literature that digital technologies are gradually shifting nurses’ roles, moving them from being the primary source of information toward a more facilitative role, in which they support parents in interpreting digital content, checking understanding, and providing personalized guidance when needed [16].

Nurses who were involved in shaping the technology, rather than simply having it introduced to them, tended to integrate it more easily and view it more positively [2,16].

Care Quality Risks

One finding from the reviewed literature stands out as a caution for policy and practice. A retrospective cohort study found that following telemedicine adoption, missed blood pressure measurements increased dramatically - from 3.9% in 2019 to 36.8% in 2023 (p < 0.0001) - with all missed measurements occurring during telemedicine visits [24]. This finding may indicate a potential patient safety concern and emphasizes the need for careful implementation of digital technologies in clinical settings.

Implementation Factors

Facilitators: Strong parental interest in digital tools was a consistent theme throughout the evidence, and this enthusiasm naturally supported uptake [3,19]. Technologies that were developed with input from nurses, clinicians, and former NICU families tended to integrate more successfully into clinical workflows [16,21].

Consulting nursing staff before adoption, rather than presenting them with a fait accompli, was consistently identified as a crucial factor in smooth implementation [14]. The near-universal availability of smartphones across income levels also made mobile applications a practically feasible option for most families [6].

Barriers: Credibility problems with commercially available apps were a recurring concern [10,23], as were technical difficulties such as firewall restrictions and scheduling conflicts in videoconferencing implementation [2]. Digital equity concerns were also frequently reported - not all parents have equal access to smartphones, reliable internet, or the digital literacy needed to engage with these tools confidently [3,9].

Among nursing staff, workload concerns were a significant barrier to adoption [10,12]. At a system level, the so-called "pilotitis" problem - where promising pilot programs fail to achieve sustained implementation - was identified as a persistent challenge, particularly for mobile app interventions [6].

Cost and sustainability: The financial picture remained largely unclear throughout the reviewed literature. Telemedicine appeared to reduce family travel time and costs [21], and a limited number of studies suggested potential cost savings through shorter NICU stays [6], but no comprehensive cost-effectiveness analyses were reported [2,12,16,17].

Sustainability was also a concern for app-based interventions that had been developed with grant funding, where ongoing maintenance and updating could not be guaranteed [23].

COVID-19 context: The pandemic accelerated telemedicine expansion at a pace that neonatal care had never previously experienced [7]. Visitation restrictions made remote communication tools essential almost overnight, and in a number of institutions, digital communication became normalized in a way that would have taken far longer under ordinary circumstances [17,20].

The retrospective cohort comparing 2019 and 2023 data showed that this rapid adoption came with trade-offs, including the care gaps described earlier [24]. The lesson here is not that pandemic-period adoption was a mistake, but that post-pandemic practice now needs to be fine-tuned, preserving what worked while reintroducing essential in-person clinical elements that were lost along the way.

Discussion

Effectiveness and the Quality of Evidence

The strongest quantitative evidence in this area comes from Wang and He’s meta-analysis [18], which reported significant reductions in maternal anxiety and improvements in hospitalization satisfaction among 1,450 parents. These findings are consistent with the broader pattern of positive parent-reported outcomes observed across qualitative and experimental studies [2,3,19,21,25].

However, the methodological quality of the evidence base remains a concern. Most primary studies were rated as low to very low quality [3,25], and common limitations - including small sample sizes, non-randomized designs, and reliance on self-reported outcomes - require cautious interpretation.

The credibility gap in commercially available mobile applications is another important finding [23]. Institution-developed technologies tend to demonstrate more consistent outcomes due to clinical oversight and alignment with care processes. In contrast, consumer-market applications may lack requirements for clinical accuracy or effectiveness. This gap highlights the need for healthcare institutions to critically evaluate applications using validated tools such as MARS before recommending them to families [23,25].

Interactive Versus Passive Technologies

A consistent pattern in the evidence is the distinction between interactive and passive technologies. Interactive tools, such as videoconferencing, messaging services, and EHR-integrated platforms, enable parents to ask questions, receive feedback, and actively participate in care. These technologies are associated with improved psychological outcomes, including reduced anxiety and increased confidence [2,17,18,21].

In contrast, passive webcam monitoring presents a more complex picture. While many parents report reassurance from visual access to their infant [12,15], others experience distress when observing difficult situations without the ability to intervene or communicate [12,14].

These findings suggest that the effectiveness of digital technologies depends not only on access but also on the level of interaction they provide. Technologies that promote parental involvement appear more beneficial, whereas passive observation alone may be insufficient and, in some cases, counterproductive. Therefore, passive systems should be complemented by accessible communication channels and appropriate parental preparation [12,14,18].

Parent and Nurse Perspectives

Another key finding is the divergence between parent and nurse experiences of digital technologies. Parents generally perceive these tools positively, as they provide connection, information, and a sense of involvement during a stressful period [9,10,16].

In contrast, nurses often report increased workload, additional communication demands, and concerns related to privacy and legal responsibility [10,12]. This difference reflects variations in how the same technologies are experienced in practice rather than disagreement about their value.

Addressing this imbalance is essential for successful implementation. Involving nurses in decision-making processes [14], providing dedicated technology support roles [12], clarifying communication expectations, and ensuring adequate staffing are key strategies. Evidence suggests that when these measures are in place, nurses’ perceptions become more positive [2,16].

Healthcare Utilization: Why Context Matters

The findings regarding healthcare utilization highlight the importance of context. Digital interventions were not associated with reduced NICU length of stay in meta-analytic evidence [18], likely because discharge timing is primarily determined by the infant’s clinical stability.

In contrast, post-discharge outcomes appear more responsive to digital interventions. Reduced emergency department visits have been reported among families receiving eHealth-supported education [22], suggesting that improved parental knowledge and confidence play a greater role once families assume primary caregiving responsibilities.

Some evidence suggests that digital tools targeting parental preparedness, such as feeding skills, medication management, and recognition of warning signs, may contribute to earlier discharge [6]. However, this finding remains limited and requires further investigation.

Equity and Access

Digital technologies are not equally accessible to all families. Barriers such as limited digital literacy, socioeconomic constraints, language differences, and restricted internet access may limit engagement [3,9].

Some interventions, such as multilingual platforms providing content in multiple languages, demonstrate how technology can be designed to improve inclusivity [16]. However, broader implementation strategies are needed.

Healthcare institutions should assess digital access and literacy during admission and ensure that digital tools complement rather than replace traditional education methods. Involving families from diverse backgrounds in the design process is essential to identify and address barriers early [3,9,21].

Limitations

Most of the included primary studies were small in scale, non-randomized, and relied on parent self-report measures, which may limit both the internal validity of individual findings and the generalizability of the results. In addition, some sources consisted of review protocols without published outcomes, and in several cases, data extraction was based on summaries or secondary sources due to the unavailability of full-text articles, potentially affecting the depth and reliability of the synthesized evidence.

As this study was conducted as a narrative review, a systematic search strategy, formal study selection process, and standardized quality appraisal (e.g., risk of bias assessment) were not applied, which may have introduced selection bias. Furthermore, the absence of a registered review protocol (e.g., PROSPERO) limits the methodological transparency and reproducibility of the review.

The restriction to English-language publications may have resulted in the exclusion of relevant studies conducted in other settings. Publication bias is also a concern, as studies reporting non-significant or negative findings are less likely to be published, potentially leading to an overestimation of the effectiveness of digital interventions. Likewise, the rapid evolution of digital health technologies means that some earlier findings may not fully reflect current practices. Finally, the use of a narrative synthesis approach, while appropriate given the diversity of the included studies, does not allow for statistical pooling of effect sizes.

Nursing Implications

This review has several important implications for clinical practice. First, nurses should not be passive recipients of technology-related decisions; rather, they should be actively involved in the evaluation, selection, and, where possible, co-design of digital parent education tools. For example, nurses can participate in institutional technology committees, contribute to usability testing of mobile applications, and provide structured feedback based on clinical workflow compatibility. Given the quality concerns identified in many consumer health applications, professional oversight is essential [10,23].

Second, digital health competencies should be recognized as a core element of neonatal intensive care nursing. In practice, this may include structured in-service training programs, integration of digital literacy modules into continuing education, and the use of standardized checklists to guide nurses in evaluating the reliability of digital content and supporting parents in its use [3].

Third, parental engagement with digital education tools should be formally integrated into the nursing care plan. This can be operationalized by documenting digital education activities in patient records, setting specific goals for parental engagement, and incorporating follow-up discussions during routine care interactions to assess understanding and usage. Fourth, healthcare institutions planning to implement digital technologies should actively consult nursing staff during the decision-making process and carefully consider workload implications. Practical strategies may include conducting workload impact assessments prior to implementation, allocating protected time for technology-related tasks, and establishing dedicated digital support roles (e.g., clinical digital facilitators) where additional burden is identified [12,14].

Finally, equity considerations must be integrated from the outset. Routine assessment of digital literacy at admission using brief screening tools, combined with tailored education strategies that integrate both digital and traditional methods, can help ensure equitable access. Providing alternative formats (e.g., printed materials or face-to-face education) and offering additional support for families with low digital literacy are essential steps. Nurses are also well-positioned to lead future research in this field, particularly in addressing current evidence gaps related to implementation costs, training needs, comparative effectiveness of digital tools, and long-term infant outcomes.

## Conclusions

Digital health technologies may help improve parental experiences in NICU settings, particularly by reducing anxiety and enhancing satisfaction, confidence, knowledge, and self-efficacy. These findings support their integration into family-centered neonatal care. However, their effectiveness depends on careful and well-structured implementation. Poorly planned adoption may introduce patient safety risks, disrupt clinical workflows, and increase nursing workload, which can negatively affect care quality. Concerns regarding the reliability of commercially available mobile applications further highlight the need for professional oversight, with clinician-guided or institution-developed tools offering safer and more reliable alternatives.

Future research should focus on high-quality study designs, standardized outcomes, and long-term evaluation of both parental and infant effects. Until stronger evidence is available, digital technologies should be used as a complement to nurse-led care rather than a replacement. The relational and personalized nature of nursing care remains essential and is unlikely to be fully replicated through digital approaches.
